# Epigenetic regulation by DNA methylation in PCOS and POI: mechanisms of ovarian dysfunction and implications for clinical research

**DOI:** 10.1186/s13048-026-02040-x

**Published:** 2026-02-23

**Authors:** Rubing Hu, Ling Jin, Qiaodan Li, Peiyin Fan, Weihong Fan, Jian Xu

**Affiliations:** 1 Center for Reproductive Medicine, the Fourth Affiliated Hospital of School of Medicine, and International School of Medicine, International Institutes of Medicine, Zhejiang University, Yiwu, 322000 China; 2 Department of Obstetrics and Gynecology, Center for Reproductive Medicine, the Fourth Affiliated Hospital of School of Medicine, and International School of Medicine, International Institutes of Medicine, Zhejiang University, Yiwu, 322000 China

**Keywords:** DNA methylation, Epigenetic, Genome-wide association studies, Ovary function, Polycystic Ovary Syndrome (PCOS), Premature Ovarian Insufficiency (POI)

## Abstract

Ovulation disorders are the leading cause of female infertility, primarily due to abnormal regulation of the hypothalamic-pituitary-ovarian (HPO) axis or dysfunction of target organs. The most common conditions in this category are polycystic ovary syndrome (PCOS) and premature ovarian insufficiency (POI), with the core mechanisms involving follicular developmental abnormalities and impaired oocyte quality. However, the pathophysiological mechanisms underlying PCOS and POI are not yet fully understood. Recent studies have shown that epigenetic regulatory mechanisms play a crucial role in the pathological processes of PCOS and POI, with DNA methylation key epigenetic modification emerging as a focal point of research. Aberrant DNA methylation regulation has been closely linked to follicular developmental disorders and endocrine dysfunction in reproduction. Although research on the role of DNA methylation in PCOS/POI pathology is still in its early stages, it has already demonstrated significant potential as a key epigenetic target. In this study, we comprehensively review the relationship between DNA methylation abnormalities and the pathogenesis of PCOS/POI, exploring their mechanisms of action. We also discuss new research directions and potential epigenetic biomarkers for treating associated comorbidities and improving the quality of life in female patients with ovarian dysfunction. This provides a theoretical foundation for advancing epigenetic diagnostic marker screening, targeted intervention strategies, and accelerating the translation of epigenetic regulatory mechanisms into clinical diagnostic and therapeutic applications.

**Graphical abstract:**

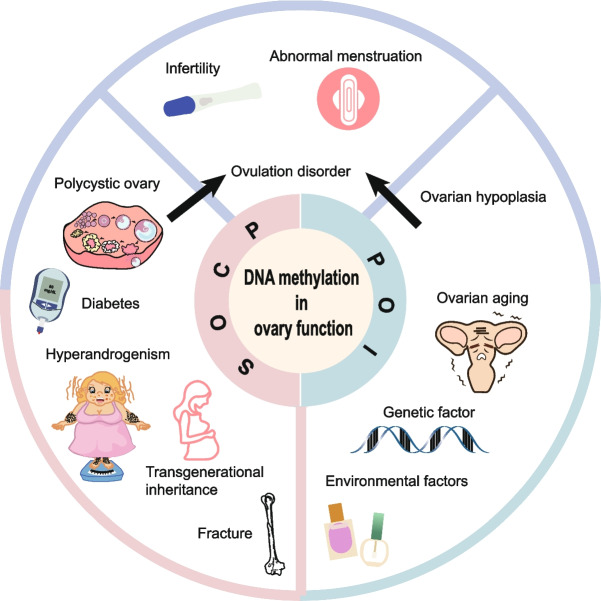

## Introduction

Reproductive health is a vital component of population development strategies. The growing challenge of population aging, declining fertility rates among individuals of reproductive age, and the increasing incidence of birth defects have become pressing concerns in reproductive medicine. The World Health Organization (WHO) defines female infertility as the inability to conceive after at least 12 months (or longer) of regular, unprotected sexual intercourse, encompassing both primary and secondary infertility [1, 2]. Currently, female infertility is recognized as a global public health issue, affecting over 186 million individuals worldwide, particularly in developing countries [3, 4]. This condition significantly diminishes the quality of life for affected women and imposes substantial public health burdens, including psychological stress, financial strain, and disruptions to family dynamics [2, 5]. Among the various contributing factors, ovarian health is critical in female fertility. In recent years, increasing attention has been directed toward understanding the relationship between DNA methylation and ovarian function. Consequently, elucidating the molecular mechanisms underlying ovarian function and the influence of DNA methylation could provide a valuable foundation for molecular diagnostics and genetic counseling in the context of female infertility.

As the primary female reproductive organ, the ovary plays a crucial role in producing oocytes to maintain fertility and in secreting sex steroid hormones that regulate endocrine function [6]. Clinically, female reproductive endocrine disorders are among the most common gynecological conditions. These disorders are primarily caused by dysfunction of the hypothalamic-pituitary-ovarian (HPO) axis or abnormalities in the response of target organs, ultimately leading to ovulatory dysfunction and menstrual irregularities [7–9]. The common reproductive endocrine disorders in women of reproductive age are PCOS [10, 11] and POI [12], both of which are closely associated with clinical symptoms such as anovulation, menstrual disturbances, and infertility [13–15]. Although POI affects approximately 1% of women under 40 and is generally considered a distinct endocrine disorder, it may also coexist with other endocrine conditions [16]. In recent years, the incidence of hormonal imbalances in women has risen sharply, largely due to increasing exposure to environmental pollutants and chemical substances [17–19]. Additionally, heightened social stress, changes in dietary patterns, and disruptions in circadian rhythms have contributed to mutations in susceptibility genes, further exacerbating the prevalence of female reproductive endocrine disorders [20–22].

This study provides an overview of DNA methylation, the most common form of epigenetic modification, and explores its underlying epigenetic mechanisms in patients with PCOS and POI. Additionally, it introduces potential epigenetic biomarkers and therapeutic targets to offer new strategies to improve ovarian function and enhance fertility in individuals affected by PCOS or POI.

### DNA methylation and the pathological mechanisms of PCOS

Several studies have demonstrated significant differences in DNA methylation patterns between ovaries affected by PCOS and those of healthy individuals [23–25]. The genomes of patients with PCOS tend to exhibit a globally hypomethylated state, which is primarily associated with dysregulation of hormonal pathways, inflammatory responses, and disturbances in lipid and glucose metabolism [26]. Similar epigenetic alterations have also been observed in the offspring of hyperandrogenic pregnant mice, supporting the heritability and developmental origins of PCOS [27]. These findings contribute to a deeper understanding of the pathological mechanisms underlying PCOS.

### Definition of DNA methylation

**Fig. 1:**
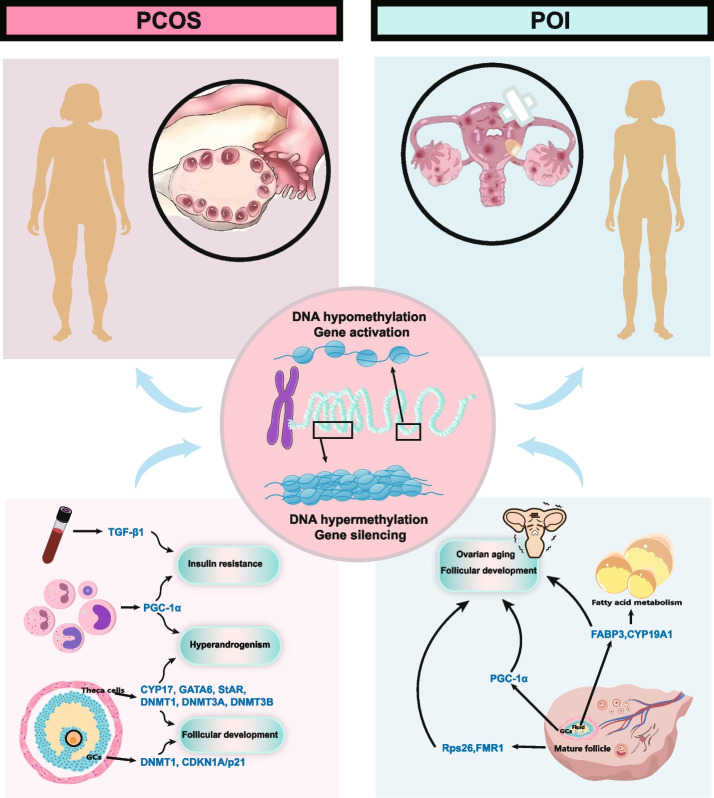
The mechanisms of DNA methylation in PCOS and POI

DNA methylation is a key epigenetic modification in mammals, characterized by the covalent addition of a methyl group to the C-5 position of cytosine residues, a process catalyzed by DNA methyltransferases (DNMTs) [28]. Generally, DNA hypermethylation in gene promoter regions is associated with transcriptional repression, whereas hypomethylation tends to enhance gene transcription (Fig. 1). DNA methylation plays an essential role in regulating numerous physiological processes, including gene expression, embryonic development, cell proliferation and differentiation, genomic stability, genomic imprinting, and chromatin remodeling [29–31]. Moreover, aberrant DNA methylation is implicated in a range of pathological conditions, including tumorigenesis and various other diseases [32].

### Polycystic ovarian syndrome (PCOS)

PCOS is one of the most common endocrine disorders affecting women of reproductive age. It is a clinical syndrome characterized by hyperandrogenism, oligo-ovulation or anovulation, and polycystic ovarian morphology [33, 34]. The global prevalence of PCOS varies depending on the diagnostic criteria used. For instance, based on the Rotterdam criteria, the global prevalence is estimated to be between 10 and 13% [35–37], whereas the National Institutes of Health (NIH) criteria estimate a prevalence of approximately 5.5% [38]. PCOS often emerges during puberty and can persist throughout a woman’s reproductive lifespan, extending into menopause [39]. It is associated with a variety of gynecological complications, including menstrual irregularities, infertility, endometrial hyperplasia, and an increased risk of endometrial cancer [40]. Furthermore, PCOS is closely linked to several metabolic disorders that severely impact women’s reproductive health and overall well-being, such as obesity, metabolic syndrome, insulin resistance, hyperinsulinemia, type 2 diabetes, dyslipidemia, and cardiovascular diseases [35]. Despite extensive global efforts, including genome-wide association studies, the genetic basis of PCOS remains largely unclear. However, increasing evidence suggests that epigenetic modifications, such as DNA methylation, histone acetylation, and microRNAs are involved in the onset and progression of PCOS [41, 42]. Collectively, these findings highlight the central role of epigenetic dysregulation in the pathogenesis of PCOS. Nevertheless, the specific mechanisms by which DNA methylation contributes to PCOS require further investigation. Therefore, this study aims to summarize current knowledge on the relationship between DNA methylation and the development of PCOS.

### DNA methylation and ovulatory disorders in PCOS

Patients with PCOS often exhibit a range of ovarian dysfunctions, including impaired follicular development, anovulation, and reduced oocyte quality, all of which pose significant threats to female reproductive health [43]. Altered DNA methylation levels in specific genes within oocytes have been observed, potentially affecting oocyte maturation, fertilization capacity, and early embryonic development. Notably, genes involved in cell cycle regulation, apoptosis, and metabolism often display aberrant DNA methylation patterns, which may contribute to oocyte dysfunction [44]. Previous studies have consistently reported that oocyte quality in women with PCOS is often compromised, resulting in lower rates of fertilization, cleavage, and implantation, as well as higher miscarriage rates [45]. They may be associated with elevated homocysteine (Hcy) levels in follicular fluid [46]. One study demonstrated that homocysteine impaired porcine oocyte quality by disrupting one-carbon metabolism and inducing hypermethylation of mitochondrial DNA, leading to mitochondrial dysfunction and reduced developmental competence. Interestingly, these adverse effects were mitigated by treatment with the DNA methyltransferase inhibitor 5-azacytidine (5-AZA) [47]. Additionally, Anti-Müllerian hormone (AMH) plays a key role in follicular development by acting through the AMH receptor type II (AMHR2) [48], and aberrant DNA methylation of AMHR2 has been closely linked to the pathogenesis of PCOS [49]. Collectively, these findings underscore the critical role of DNA methylation in regulating follicular development and oocyte quality in PCOS.

Granulosa cells (GCs) within ovarian follicles play a critical role in providing nutrients and growth factors necessary for oocyte development, thereby supporting follicular maturation and ovulation. Impaired proliferation of GCs is widely recognized as a key contributor to follicular maturation disorders. DNA methylation profiling of GCs in patients with PCOS has revealed epigenetic alterations in genes involved in the regulation of essential ovarian functions [44]. Notably, inhibition of DNA methyltransferase 1 (DNMT1) has been shown to modulate the expression of the cyclin-dependent kinase inhibitor 1 A (CDKN1A/p21), thereby suppressing GC proliferation and inducing cell cycle arrest at the G0/G1 phase in PCOS patients [50]. This mechanism may underlie, at least in part, the follicular development abnormalities observed in PCOS. Moreover, hypomethylation of promoter regions in genes associated with lipid and steroid biosynthesis in GCs has been linked to their aberrant overexpression, promoting excessive steroid hormone production, particularly androgens. These findings offer novel insights into the molecular basis of hyperandrogenemia in PCOS [51, 52].

### DNA methylation changes in theca cells

Theca cells are the primary source of androgen synthesis in the ovary, and their functional abnormalities are closely linked to the pathogenesis of PCOS. Although research on DNA methylation in theca cells remains relatively scarce, some evidence indicates that aberrant DNA methylation may contribute to disease development by modulating the expression of steroidogenic genes. These findings provide important insights into the molecular mechanisms underlying hyperandrogenism and follicular dysfunction in PCOS.

In prenatally androgenized rat models, a mild global reduction in promoter methylation of steroidogenic genes, including CYP17, GATA6, and StAR, has been observed in theca cells. These alterations in promoter methylation may contribute to PCOS-associated hyperandrogenism by enhancing the expression of steroidogenic genes [53]. Similarly, significant hypomethylation at specific CpG sites within the *Follistatin* promoter, accompanied by increased transcription, further supports the notion that aberrant DNA methylation in theca cells modulates key reproductive factors. Such epigenetic dysregulation may influence steroid biosynthesis and follicular development, thereby providing a mechanistic basis for PCOS-like phenotypes [54]. Furthermore, in a prenatally androgen-exposed sheep model, abnormal expression of DNA methylation-related enzymes (DNMT1, DNMT3A, DNMT3B) and demethylation enzymes (TET family) was detected in theca cells, reflecting cell type-specific epigenetic imbalance. Collectively, these findings indicate that disrupted DNA methylation may impair the transcriptional regulation of steroidogenic genes, leading to defective follicular development and hyperandrogenism. This highlights DNA methylation dysregulation as a key molecular mechanism through which prenatal androgen exposure mediates follicular dysfunction [55].

In summary, these studies collectively reveal that DNA methylation in theca cells and its associated epigenetic regulation play a central role in ovarian dysfunction induced by prenatal androgen exposure. They provide crucial theoretical foundations for further elucidating the molecular mechanisms of PCOS and identifying potential therapeutic targets.

### DNA methylation and insulin resistance (IR) in PCOS

DNA methylation imbalances in patients with PCOS are closely associated with IR, and these epigenetic alterations may contribute significantly to the onset and progression of PCOS by regulating gene expression, ultimately leading to elevated androgen levels and impaired insulin sensitivity. Approximately 50%−75% of women with PCOS exhibit IR [56]. In one study, researchers identified the susceptibility gene *TGF-β1* in peripheral blood samples of PCOS patients with IR. Hypomethylation at the CpG4 and CpG7 loci of this gene was found to potentially alter its expression, thereby playing a critical role in the pathogenesis of insulin resistance in PCOS [57]. Another study reported increased DNA methylation in the promoter region of peroxisome proliferator-activated receptor gamma coactivator 1-alpha (PGC-1α, also known as PPARGC1A) in leukocytes from PCOS patients, which was associated with both IR and elevated androgen levels [58]. Furthermore, DNA methylation analyses of subcutaneous adipose tissue revealed distinct epigenetic patterns based on obesity status. Non-obese PCOS women showed decreased methylation at the luteinizing hormone/choriogonadotropin receptor (LHCGR) locus, while obese PCOS women exhibited increased methylation at the insulin receptor (INSR) locus. These methylation changes were linked to dysregulated expression of *LHCGR* and *INSR*, contributing to increased ovarian androgen production and peripheral insulin resistance, respectively [59, 60].

Overweight and obesity play a key role in the development of IR, especially visceral obesity, which is characterized by adipose tissue mass and dysfunctional adipose tissue [61]. It is now widely recognized that adipose tissue dysfunction in PCOS is largely driven by the exposure of adipocytes to elevated androgen levels [62, 63], which subsequently leads to the release of adipocytokines with deleterious metabolic effects [63]. In women with PCOS, hypomethylation of promoter regions in genes involved in lipid and steroid biosynthesis has been observed, resulting in the upregulation of steroid hormone production [52]. Furthermore, both clinical studies and animal models of PCOS have demonstrated a strong association between hyperandrogenemia (HA) and white adipose tissue (WAT) dysfunction [64]. Interestingly, low-frequency electroacupuncture has been shown to significantly alter DNA methylation patterns in subcutaneous adipose tissue, thereby improving systemic metabolic function in PCOS patients [65].

Defects in skeletal muscle function can significantly disrupt metabolic homeostasis and contribute to the development of IR. Genome-wide analyses of skeletal muscle tissue from women with PCOS have revealed that the overexpression of certain genes is associated with specific alterations in DNA methylation, which may influence metabolic processes by modulating gene expression [66]. One of the key mechanisms underlying skeletal muscle IR in PCOS appears to involve disruptions in intracellular insulin signaling pathways, mitochondrial dysfunction, impaired fatty acid oxidation, and reduced levels of lipocalin [67, 68]. However, current research on the role of DNA methylation in skeletal muscle IR remains limited [68], highlighting the need for further in-depth investigation in this area.

### DNA methylation and transgenerational inheritance of PCOS

PCOS is believed to have a heritable component, and increasing evidence suggests that epigenetic modifications, particularly DNA methylation, may play a role in its transgenerational transmission. For instance, reproductive and metabolic abnormalities have been observed in third-generation female offspring following AMH exposure during gestation in mice. These effects were associated with altered DNA methylation profiles, which mirrored the hypomethylation patterns found in both women with PCOS and their daughters [69, 70]. Both clinical and experimental data support the idea that the intrauterine environment in PCOS affects DNA methylation patterns in the promoter regions of key genes involved in reproduction and metabolism. This indicates that epigenetic modifications triggered by environmental factors during pregnancy may contribute to the development of PCOS in offspring [71]. Collectively, these findings suggest that PCOS phenotypes can be epigenetically inherited via DNA methylation, and that such methylation markers hold potential as diagnostic tools for early detection and risk assessment of PCOS.

### DNA methylation and intestinal microbial factors of PCOS

Previous study showed that specific bacterial genera and metabolites are significantly altered in PCOS patients, and there are complex associations with clinical indicators [72]. Environmental and metabolic factors such as intestinal flora dysbiosis, bile acid metabolism disorders, and poor diet play an important role in the pathogenesis of PCOS. In addition, previous studies has shown that bile acids, as a key metabolic regulator, whose synthesis and conversion are regulated by the intestinal flora, can affect hormonal balance and metabolic homeostasis, and that intestinal microecological disorders promote endocrine and metabolic abnormalities through multiple pathways, which are involved in the occurrence and progression of PCOS [73]. From a therapeutic point of view, PCOS should be treated with multidimensional interventions, including a healthy diet and exercise management, as well as interventions targeting intestinal flora and bile acid metabolism, in order to alleviate the symptoms of PCOS and improve metabolic health. Future studies need to further elucidate the complex association between the Gut-Bile-Metabolism axis.

### Prospects of DNA methylation in the diagnosis and treatment of PCOS

The clinical manifestations of PCOS are highly heterogeneous, and the lack of unified diagnostic criteria continues to hinder accurate and early diagnosis. Future improvements in diagnostic precision may be achieved through the identification and application of reliable biomarkers and standardized diagnostic protocols. In recent years, growing evidence has highlighted the significance of DNA methylation in the diagnosis and treatment of PCOS. For example, studies have demonstrated that the methylation profiles of three specific CpG sites in peripheral blood can accurately distinguish PCOS patients from healthy individuals [74]. Additionally, a diagnostic model based on methylation markers developed using LASSO regression has shown exceptionally high diagnostic accuracy [75], providing strong support for the use of DNA methylation as a clinical diagnostic tool.

Emerging research also suggests therapeutic potential in targeting DNA methylation to prevent or mitigate PCOS. One study found that caloric restriction can prevent the transgenerational inheritance of PCOS by reprogramming DNA methylation in oocytes, emphasizing the critical role of preconception management [76]. Moreover, PCOS is often associated with depressive-like behaviors, which are linked to elevated expression of DNA methyltransferases in the prefrontal cortex and hippocampus. Acetate treatment was shown to alleviate these symptoms by inhibiting DNA methylation [77]. In addition, low-frequency electroacupuncture targeting skeletal muscle has demonstrated considerable therapeutic efficacy in improving metabolic dysfunction in PCOS patients, particularly for those unable to engage in conventional physical activity [78]. Taken together, these findings underscore the promising potential of both physical and pharmacological interventions targeting DNA methylation pathways in the future treatment of PCOS.

### DNA methylation and POI

Proper epigenetic modifications during follicular development are critical for normal oocyte growth and maturation. The pathogenesis of POI primarily involves a diminished primordial follicle reserve, accelerated follicular atresia, and impaired follicular development [79]. Recent studies have identified specific genome-wide DNA methylation abnormalities in the peripheral blood of patients with POI. These epigenetic alterations are closely linked to the clinical manifestations of POI and its long-term complications, including cardiovascular disease and osteoporosis [80]. Aberrant DNA methylation significantly impacts key biological pathways, such as hormone metabolism, follicular development, lipid metabolism, immune regulation, and cardiovascular and bone homeostasis. Emerging evidence suggests that aberrant DNA methylation plays a central role in the pathogenesis of POI by disrupting multiple physiological processes. These findings provide novel insights into its underlying mechanisms and underscore the potential of epigenetically targeted therapeutic strategies [81].

### Primary ovarian insufficiency (POI)

Primary ovarian insufficiency (POI) constitutes a significant threat to female reproductive health and is a leading cause of infertility among women of reproductive age [82]. POI is defined as progressive ovarian dysfunction occurring before the age of 40, characterized by clinical manifestations such as menstrual irregularities (including oligomenorrhea or amenorrhea), elevated gonadotropin levels (follicle-stimulating hormone, FSH > 25 IU/L), and reduced estradiol concentrations [82], the advanced stage of this disorder is referred to as premature ovarian failure (POF) [83]. In both clinical practice and academic research, POI and POF are generally considered different stages along the same disease spectrum, with some literature using the terms interchangeably to describe ovarian insufficiency before the age of 40 [13]. Over the past two decades, the prevalence of POI has increased markedly. Based on the diagnostic criteria of the European Society of Human Reproduction and Embryology (ESHRE), its global prevalence was estimated at approximately 3.5% in 2023 [84, 85]. The etiology and pathogenesis of POI are complex and highly heterogeneous. Contributing factors include genetic abnormalities (with chromosomal defects accounting for ~ 15%), autoimmune disorders, infections, iatrogenic causes such as chemotherapy or radiotherapy, and environmental exposures. Nevertheless, approximately 70% of cases are classified as idiopathic, with the underlying cause remaining undefined [80].

### DNA methylation and ovarian aging

In female mammals, aging is closely associated with the depletion of the ovarian follicular reserve [86], and DNA methylation is a hallmark of mammalian aging [87]. Disruptions in DNA methylation are frequently linked to various reproductive disorders and play a pivotal regulatory role in oogenesis and oocyte maturation [88]. DNA methylation contributes to the maintenance of ovarian function by regulating gene silencing, transcriptional repression, and chromatin remodeling. Aberrant methylation can lead to granulosa cell apoptosis, reduced follicle numbers, and hormonal imbalances, all of which are characteristic features of POI. Members of the mammalian DNA methyltransferase (DNMT) family, including DNMT1, DNMT2, DNMT3A, DNMT3B, and DNMT3L have distinct roles and mediate crucial epigenetic modifications. These enzymes are essential in regulating cellular senescence and reproductive aging by repressing the transcription of target genes [88]. Both human and mouse studies have demonstrated that DNMT1 expression is downregulated in GCs during ovarian aging, leading to hypomethylation of the *TP53* promoter region and thereby accelerating ovarian senescence [89]. Additionally, research has shown that the expression of DNMT1, DNMT3A, DNMT3B, and DNMT3L is significantly reduced in oocytes of aged female mice, accompanied by global hypomethylation, suggesting that epigenetic imbalance may be a major contributor to declining reproductive potential [90]. Notably, both the transcriptome and DNA methylome of GCs exhibit marked, non-random alterations during the decline of ovarian function. Age-related downregulation of gene expression predominantly occurs in hypomethylated regions within gene bodies rather than in traditional promoter regions, uncovering a potential novel mechanism of epigenetic regulation in ovarian aging [91].

### DNA methylation in follicular development

Animal studies have demonstrated that excessive sodium fluoride (NaF) exposure elevates DNA methylation levels and suppresses the expression of the maternally imprinted gene *Nnat*. This suppression impairs glucose transport and disrupts oocyte metabolism, ultimately hindering follicular development and diminishing ovarian reserve function [92]. Another study reported that deletion of *Rps26* in mouse oocytes led to decreased DNA methylation and disrupted chromatin transition from the non-surrounded nucleolus (NSN) to surrounded nucleolus (SN) configuration, resulting in oocyte apoptosis and follicular atresia [93]. GCs play a pivotal role in establishing the primordial follicle pool and are essential for maintaining female reproductive health and fertility [94]. The development of primary ovarian insufficiency (POI) is strongly associated with GC apoptosis, often triggered by oxidative stress, which is a common cause of follicular atresia [95]. Fatty acid-binding proteins (FABPs), a family of low molecular weight proteins, are widely expressed across tissues and organs and are involved in intracellular fatty acid transport and regulation [96]. Genome-wide DNA methylation profiling has identified 11 hypermethylated gene promoters in POI patients, with significant downregulation in mRNA expression of six genes, notably *FABP3*, which showed the most pronounced decrease. *FABP3* is implicated in the regulation of estradiol synthesis via the granulosa cell-specific enzyme *CYP19A1*, and its downregulation may disrupt fatty acid metabolism in follicles, highlighting a potential pathophysiological mechanism in POI [97]. Study has shown that the level of AMH expression is significantly reduced in patients with poor ovarian functional response and abnormal CpG island methylation near their transcription termination site (TES), suggesting that epigenetic regulation may be involved in the mechanism of ovarian dysfunction through the inhibition of AMH expression [91].

### DNA methylation and genetic/environmental factors

Abnormal epigenetic regulation of genes involved in ovarian function may contribute to follicular dysfunction and diminished ovarian reserve by altering DNA methylation or histone modification patterns, ultimately leading to the development of POI. Turner syndrome (45, monosomy X) and Fragile X syndrome are among the most common genetic etiologies associated with POI [80]. In Turner syndrome, genome-wide DNA methylation analysis has revealed a pattern of global hypomethylation alongside localized hypermethylation, suggesting that epigenetic alterations play a role in disease phenotype formation by modulating gene expression [98]. Studies have also reported widespread DNA methylation abnormalities in the blood of Turner syndrome patients, indicating the possibility of epigenetic dysregulation in ovarian tissues [99]. The *FMR1* gene (Fragile X Mental Retardation 1), which is critically involved in Fragile X syndrome (FXS), is highly expressed in female GCs and plays a key role in regulating folliculogenesis efficiency. Its transcriptional activity is modulated by the methylation status of CpG islands within its promoter region [100]. Notably, significant differences in methylation at the CpG 94 locus in primary granulosa cells have been observed between women with normal and diminished folliculogenesis efficiency, suggesting that dynamic epigenetic regulation of FMR1 may influence follicular development [101].

Primordial germ cells (PGCs) represent the earliest stage of follicular development and are particularly susceptible to environmental influences. Environmental chemicals can disrupt normal epigenetic programming, thereby impairing germ cell development [102]. Animal studies have demonstrated that exposure to environmental pollutants can induce changes in DNA methylation, impair ovarian function, and if these epigenetic alterations are stably maintained in the germline, facilitate transgenerational inheritance of reproductive dysfunction [103]. One study investigated the transgenerational epigenetic effects of environmental toxicant mixtures (e.g., fungicides, insecticides, plastics) and found that brief exposure of F0 generation females during pregnancy led to ovarian disease phenotypes, such as PCOS and reduced ovarian reserve, in both F1 and F3 generation adult rats. Altered DNA methylation and transcriptome profiles were detected in granulosa cells of the F3 generation, highlighting that environmental exposures can induce ovarian disease through heritable epigenetic mechanisms [104, 105].

### Prospects of DNA methylation in the diagnosis and treatment of POI

In recent years, aberrant DNA methylation has been increasingly recognized as a key molecular mechanism contributing to the pathogenesis of POI/POF. Unlike traditional gene mutations, patients with POI/POF frequently exhibit widespread epigenetic alterations, particularly disrupted DNA methylation patterns. Genome-wide methylation profiling has identified numerous differentially methylated regions in POF patients, some of which are closely linked to the transcriptional dysregulation of genes critical for ovarian function, suggesting their potential utility as diagnostic and predictive biomarkers [81]. Moreover, multi-omics studies have revealed extensive DNA methylation abnormalities in the cumulus cells of POI patients. These alterations can affect essential signaling pathways and disrupt the oocyte microenvironment, thereby accelerating ovarian functional decline. Collectively, these epigenetic insights not only deepen our understanding of disease mechanisms but also provide valuable evidence for early diagnosis and biomarker discovery [106].

Currently, the clinical management of POI/POF relies primarily on hormone replacement therapy (HRT). While HRT effectively alleviates symptoms associated with estrogen deficiency, such as vaginal dryness, hot flashes, and urogenital atrophy [107], it does not reverse ovarian decline or restore follicular development and ovulatory function. This limitation underscores the urgent need for more fundamental therapeutic strategies. Accordingly, elucidating the molecular mechanisms underlying POI/POF is essential for developing novel treatments targeting key pathogenic pathways. Animal studies have shown that modulating DNA methylation through epigenetic drugs can improve ovarian cell function and delay follicular apoptosis. For example, mildly reducing global DNA methylation in bone marrow mesenchymal stem cells (BM-MSCs) with low-dose DNMTs inhibitor (5-Aza-dC) has been reported to preserve MSC viability and specific phenotypes in POI/POF mouse models. This intervention markedly improved ovarian hormone levels and follicular development, suggesting that DNA methylation regulation may enhance the therapeutic potential of stem cell-based approaches for restoring ovarian function [108].

In summary, DNA methylation plays a pivotal role in the onset and progression of POF, and its potential as a biomarker and therapeutic target is increasingly being revealed. In the future, by integrating multi-omics data with epigenetic intervention strategies, DNA methylation holds promise for advancing precision diagnostics and innovative treatment approaches for POI/POF.

### Limitations

Existing literature highlights the potential role of DNA methylation in both PCOS and POI; however, several limitations remain, with the two conditions showing both shared and distinct challenges. Common limitations include small sample sizes, restricted study populations and tissue sources, predominantly cross-sectional designs that preclude causal inference, and inconsistent detection platforms or analytical workflows, which hinder comparability across studies. In addition, inadequate adjustment for confounding factors, such as age, BMI, metabolic status, medication use, and environmental exposures and the tendency for studies to remain at the discovery stage of differentially methylated sites without functional validation or multi-omics integration further limit interpretability. Condition-specific differences are also evident. PCOS research is relatively abundant but relies heavily on peripheral blood samples, with results often confounded by complex phenotypes and metabolic factors, while subtype-specific features remain poorly defined. In contrast, POI studies are scarce and primarily focused on cumulus or granulosa cells, lacking systematic methylation mapping across tissues and developmental stages. Moreover, the interplay among environmental exposures, immune factors, genetic susceptibility, and DNA methylation remains largely unexplored.

Additionally, although this review adopted a structured search strategy and drew upon the existing literature, its non-systematic nature may have limited the breadth and depth of retrieval. For example, gray literature, conference abstracts, and non-English publications were not comprehensively covered, which may have led to the omission of some relevant findings. Nevertheless, the included studies sufficiently capture the major recent advances in epigenetic modifications, particularly DNA methylation in PCOS and POI research, thereby providing a valuable reference for future investigations.

### Future perspectives

Future research should prioritize uncovering the shared regulatory mechanisms and distinct DNA methylation patterns underlying PCOS and POI. Integrating multi-omics technologies with clinical translational strategies will be essential for gaining a comprehensive understanding of the epigenetic mechanisms involved. Mechanistically, it is critical to elucidate how aberrant methylation of key genes drives the core pathologies of these disorders, particularly impaired GCs and abnormal follicular development. On the translational front, efforts should focus on developing invasive diagnostic tools based on DNA methylation markers in peripheral blood or follicular fluid, such as the DNA methylation clock, as well as exploring the combined therapeutic potential of targeted demethylating agents and metabolic interventions. Attention should also be given to the effects of intrauterine programming and endocrine-disrupting chemicals on transgenerational epigenetic inheritance. Moreover, addressing challenges such as sample heterogeneity and establishing causality is essential. Constructing cross-disease epigenetic regulatory networks will further advance precision diagnostics and regenerative medicine, offering innovative strategies to enhance women’s reproductive health.

## Conclusion

Abnormal DNA methylation plays a central role in the pathogenesis of PCOS and POI. In PCOS, genetic factors lead to genome-wide hypomethylation, which is associated with hyperandrogenemia and IR, exacerbating metabolic disorders through the hypomethylation of androgen synthesis genes. In contrast, POI is characterized by the inhibition of estradiol synthesis, impaired follicular development, and ovarian senescence, with GCs apoptosis. In synergy with Turner syndrome, POI alters the methylation patterns of non-coding RNAs. In terms of transgenerational inheritance, maternal AMH-mediated DNA methylation abnormalities can be transmitted to offspring via oocytes, while environmental toxins such as DEHP induce transgenerational ovarian defects by triggering methylation disorders in oocytes. These disruptions contribute to the hyperandrogenic phenotype seen in PCOS and the decline in ovarian function observed in POI. Therapeutically, demethylating drugs, caloric restriction, and electroacupuncture have been shown to reverse methylation disorders, offering potential new directions for targeted interventions. These findings underscore the central role of epigenetic dysregulation in ovarian diseases and highlight the complex interactions between genetics, the environment, and transgenerational transmission.

**Fig. 2:**
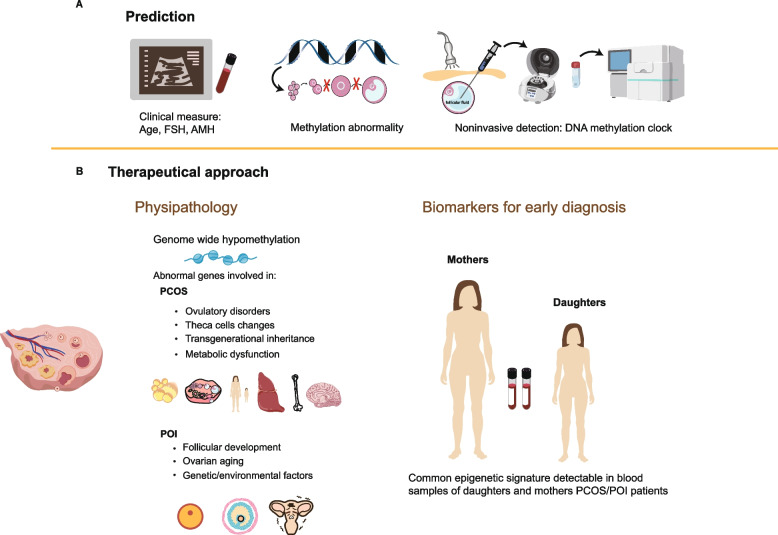
The prospects in clinical application

In recent years, accumulating data have revealed that DNA methylation abnormalities are present in women with PCOS and POI. In the future, identifying specific epigenetic changes could aid in the early detection of these disorders, potentially starting from puberty, ensuring timely diagnosis and appropriate healthcare. Therefore, epigenetic changes represent promising targets for future therapies (Fig. 2).

## Data Availability

No datasets were generated or analysed during the current study.
